# On the Treatment of Fever by Cold Water

**Published:** 1848-06

**Authors:** 


*On the Treatment of Fever by Cold Water.*—By William Gill, M. D. Physician to the Nottingham Dispensary, &c.

[In our last volume, p. 3, the reader will find a communication by Mr. Stallard, of Leicester, upon the efficacy of the external application of cold water as a refrigerant and sudorific in fever; we continue the subject by the following abstract of a paper which was read at the last meeting of the Provincial Medical and Surgical Association.]

Before entering more immediately on the subject of this paper, the author describes concisely the general features of the prevalent fever. In most cases the *immediate* cause of the attack was traceable to sleeping in crowded lodging-houses, the usual abode of fever in large cities, the proximate causes, doubtless, were over-fatigue, and insufficient and unwholesome food. The term "hunger pestilence" has been aptly applied to that disease. A true typhoid gastro-enterite was present in many patients, closely resembling what so frequently is observed in the Parisian hospitals. Whether the essentiality of the fever existed in the condition of the muco-alimentary membrane or not, it was not the author's intention to discuss. This, however, he remarked, that *so soon* as the signs of gastro-alimentary irritation were subdued, the signs of general fever subsided. Some two or three cases, which he read, corroborate this observation. In the generality of patients under his care, not only was the gastro-alimentary membrane affected, but also the muco-pulmonary, as evidenced by cough, shortness of respiration, and frequently universal sonorous râles, affecting the whole of the chest. This was not remarked among the English cases. There was discharge of blood from the inner membranes. Œdema of the lower extremities occuring early in the disease was generally a fatal symptom, though we had two cases of recovery in boys, who were universally anasarcous from the commencement. The disturbance of the sensorium was marked by a low muttering delirium, sometimes wandering about the bedroom, constant picking at the bedclothes, and subsultus tendinum.

Some were affected with a heavy, comatose, and stupid state, from which they were with difficulty aroused, and when aroused, with difficulty were made to understand questions; they relapsed immediately into the same lethargic condition when left to themselves. This comatose condition often continued till convalescence was established, and in some even later. It seemed a perect prostration of all mental energy, and was only relieved as the bodily powers regained their tone. In no one case did active delirium occur. The secretions from the bowels, were thin, frequent, and offensive, and often attended with severe griping but no bloody discharges. The function of bladder in one or two individuals was suspended, and it was necessary twice daily to use the catheter. The usual period of the termination of the fever seemed to be from the eighteenth to the twenty-first day, at which time the patients were left in a state of the greatest prostration. When the case terminated fatally, an unrousable, unconscious coma closed the scene. The usual symptoms of fever were generally present—as the hot dry skin, black tongue, urgent thirst, pulse varying from 90 to 130, insomnia, and pains in the head, back, and limbs, &c. After this brief description of the general features of the disease, he proceeeds to the treatment.

He remarks that he is well aware that a great prejudice exists in the profession against the treatment to be advocated, partly because it is opposed to preconceived opinions, and chiefly from the unprofessional manner in which it has been ushered into notice. Feeling certain, however, that he was addressing a body of gentlemen willing to receive *truth* for the *sake of itself*, he, with perfect confidence, detailed a treatment of fever as yet untaught in the schools, and generally unrecognised by the profession.

Dr. Currie, of Liverpool, was the first scientific English physician, who enlisted cold water as an external remedial agent in the treatment of fevers. Successful as the practice was under his direction, it has been little followed in later times. It is only within the last few years that the prejudice which existed against the internal and external use of water has begun to subside. "Perhaps," observes the author, "the prominence of the sanatary questions, and the many evils proved to arise from the want of a due supply of pure water, has had much to do in removing this groundless prejudice, and may have produced an undue reaction in its favor, causing it to be considered *not only* as necessary to a healthy condition, but as a *curative agent* of universal efficacy. Hence, perhaps, the public mind has been somewhat prepared to receive the hydropathic theory with much more favour than its intrinsic merits demand. An universal remedy will ever find many advocates, and, in a numerous profession like ours, there are ever men to be found who, from selfish motives, will pander to this diseased taste of the public mind. We, as an association, must ever protest against such exclusive theories as prevail in our days, being in our opinion unscientific, opposed to experience, and calculated to lead to incorrect views respecting the power of many known and valued medicinal agents. In making this protest against any exclusive theory for the cure of diseases we must not rush into the opposite extreme, and, from disbelief of their universal efficacy, deny their particular efficacy, when the touch-stone of experience speaks to the contrary."

The plan the author has adopted for the cure of fever, has been a modification of Dr. Currie's. Instead of pouring buckets of cold water over the body, he has it enveloped in a wetted sheet, an instrument more effective than Currie's in reducing the temperature of the body, and producing a warm and comfortable perspiration, which did not uniformly follow his plan. The fear of evil consequences from this treatment is groundless. He gives no opinion as to its utility, except in cases of fever. Here, however, he states that he can speak with confidence. When the skin is burning hot, and the mouth and tongue parched, the application of a sheet wrung out of cold water, and applied *closely* to the whole surface of the body, and evaporation prevented by the application of three or four blankets placed over it, produces a most grateful feeling of refreshment, which is soon followed by a more or less warm perspiration. In young people, this perspiration breaks out in from five to ten minutes after its application; in middle-aged persons the period is longer, Many uncomfortable sensations are soon relieved by its use: such as the muscular pains in the back, thighs, and legs, and the sense of aching and weariness; the thirst often becomes less, and even the dry tongue sympathises with the relaxing influence induced on the cutaneous surface. He has seen the low moaning delirium subside whilst under its use; and some patients, who have not slept before, doze, especially if the hair has previously been cut short, and a flannel night-cap wetted with vinegar and water, been applied to the head.

The simple plan he has followed has been this:—On a flock bed he has placed from three to five blankets, superimposed over these, a sheet wrung out of cold water; on which the patient, stripped, is placed, with legs outstretched, and arms to the side; the sheet is then drawn tightly around, up to the neck, and enclosing the feet; first, one blanket, then another, and so on to the whole number, are tightly drawn over the sheet, so as to have the *whole body well and closely packed.* In this state, the patient lies from a quarter of an hour to one or two hours, according to the object in view, and the effect produced. Some get tired at the end of half an hour, some can continue for one or two hours and feel very comfortable. As soon as a gentle perspiration commences, a wineglass full of water is given frequently. At the commencment of this treatment, in a case of fever, he has generally ordered its use for one hour; after that time the wet things are removed, and the sick person is placed in a bed, well wrapped in three blankets, and allowed to perspire for three hours; afterwards, the blankets are to be carefully removed, one at a time, so as to allow the perspiration to subside gradually, and the patient is then placed in bed, between the sheets.

During the whole of this period, small quantities of water, should be given. In the summer, during this process, a free ventilation may be allowed in the chamber, in winter it is necessary to have a good fire, and to have one blanket well warmed, to apply round the body, as soon as removed from the wet sheet.

Several cases of incipient fever have lost all traces of disease after the first application. If the fever be not reduced, the next day the same plan must be repeated, keeping the patient in the wetted sheet from half an hour to one hour, according to the intensity of the symptoms, and in the blankets for one or two hours. This may be repeated every day till indications of *a cool skin* arise, and then it must be immediately discontinued.

During one period of this treatment, the temperature of the atmosphere being very high, (75° to 78° in the shade,) the author has not found it advisable to keep the patient as long as two hours sweating in the blankets; from half an hour to one hour was sufficient. A longer period caused the pulse to be accelerated instead of lowered, which latter is the usual effect of the treatment, In very hot weather, when a free perspiration has been induced at the commencement of the fever, he has adopted the following plan. To wrap the sick person, for half an hour in the wet sheet, covered lightly with one blanket; to be then washed all over with a towel wetted with tepid water, then rubbed dry, and placed in bed between the sheets. He has not found it necessary to make use of this treatment more than five times to the same individual; generally, after the third or fourth application, the skin becomes cooler, and the other signs of fever gradually subside. When the skin becomes cool, and the tongue less dry, he has instantly discontinued all water remedies, and given bark, wine and broths, and it surprising how soon convalescence and strength became established. During the whole course of the fever, milk and water, or weak broths, were allowed *ad libitum*. In one person, twice in the course of the same day, owing to the intensity of the fever, it was found necessary to repeat the wet sheet, using it the second for only half the period of the first; a comfortable night ensued.

Without doubt this is the most effective mode of *quickly* reducing the temperature of the body; an equilibrium is soon established between the cold of the water and the heat of the body, and the patient becomes bathed in a natural vapour-bath, as may be felt by placing the hand under the bedclothes, Where the fever runs high, and the delirium is violent, the wet sheet may be safely applied for short periods (two minutes,) several times in the course of the day. This will be found a more effectual mode of reduding the cerebral excitement, than any other means with which we are acquainted. This refrigerating plan, used fourteen minutes, during an evening exacerbation, will often produce a few hours' refreshing sleep

The author confesses that he had, at first, great doubts as to the *safety* of this treatment, where the mucous membranes of the bronchi and gastro-alimentary passages were complicated. Very soon his fears on this head were dissipated by the convincing evidence of experience; in fact, *these* proved the cases in which the decided benefit of the treatment was most marked. The quick and embarrassed respiration, dry cough, and sonorous râles, subsided quickly after one or two applications of the wet sheet; the cough became looser, the râles moister, and expectoration was established.

The same happy change also occurred where the gastro-alimentary membranes were disordered. Generally, the first wet sheet puts a stop to the diarrhœa, and soon afterwards, pain and swelling disappeared. A confined state of the bowels was frequently the effect of the wet sheet, and it was foud necessary, in several of the patients, to resort to small doses of castor oil. In three or four cases, the symptoms of gastric and abdominal irritation or inflamation were so violent as to have justified the use of leeches, calomel, and opium: and, indeed, we know that depletion by leeches is the usual treatment followed in the Parisian hospitals, and yet by the simple means mentioned, in three days every bad symptom had vanished. A great saving is made to the patient's strength, when we can dispense with the abstraction of blood.—*Ranking's Abstract.*

